# Ventilator-associated pneumonia in males and females: A post hoc analysis of the prospective multicenter multinational EUVAP cohort

**DOI:** 10.1016/j.jointm.2026.04.008

**Published:** 2026-06-16

**Authors:** Nicolas Garin, Despoina Koulenti, Virginie Prendki, Jordi Rello

**Affiliations:** 1 Division of Internal Medicine, Riviera Chablais Hospital, Rennaz, Switzerland; 2 Division of General Internal Medicine, Geneva University Hospitals, Geneva, Switzerland; 3 Faculty of Medicine, University of Geneva, Geneva, Switzerland; 4 Department of Critical Care, King’s College Hospital Foundation Trust, London, United Kingdom; 5 UQCCR, Faculty of Medicine, The University of Queensland, Brisbane, Australia; 6 School of Medicine, University of Thessaly, Larissa, Greece; 7 School of Health Sciences, University of Ioannina, Ioannina, Greece; 8 Division of Internal Medicine for the Aged, Geneva University Hospitals, Geneva, Switzerland; 9 Division of Anaesthesia Critical Care, Pain and Emergency Medicine, IMAGINE, Nîmes University Hospital, UR-UM 107, University of Montpellier, Nîmes, France; 10 Facultad de Medicina y Ciencias de la Salud, Universitat Internacional de Catalunya, Barcelona, Spain; 11 Clinical Research Pneumonia and Sepsis (CRIPS), Research Group-Vall d’Hebron Institute Research (VHIR), Barcelona, Spain; 12 Centro de Investigación Biomédica en Red de Enfermedades Respiratorias (CIBERES), Instituto Salud Carlos III, Madrid, Spain; 13 European Study Group of Infections in the Elderly (ESGIE), European Society of Clinical Microbiology and Infectious Diseases, Basel, Switzerland; 14 Department of Medicine, University of Thessaly, Larissa, Greece

**Keywords:** Hospital-acquired pneumonia, Nosocomial infections, Sex factors, Respiratory infections

## Abstract

**Background:**

Sex is a major cause of heterogeneity in the epidemiology, management, response to treatment, and outcome of infectious and respiratory diseases. We aimed to describe differences between males and females in characteristics, management, and mortality among patients affected by ventilator-associated pneumonia (VAP).

**Methods:**

We performed a post hoc exploratory analysis of the EUVAP/CAP cohort, a prospective multinational cohort study of patients requiring mechanical ventilation in 27 intensive care units from nine countries. Sexes were compared with respect to baseline characteristics, interventions, and outcomes in univariate and adjusted analyses.

**Results:**

Among the 2436 intubated adult patients in the cohort, 450 (18.5%) developed VAP; of these VAP cases, 293 (65.1%) were male, and the median age was 64 years. Compared with females, males had more comorbidities, and underwent bronchoscopy more frequently (20.5% *vs.* 11.5%, *P*=0.016) and triple antibiotic therapy (33.1% *vs.* 22.3%, *P*=0.016)., Intensive care unit mortality was 41.3% *vs.* 45.2% (*P*=0.422). In multivariate analysis, age in years (adjusted odds ratio [aOR]=1.02, 95%confidence interval [CI]: 1.00 to 1.04), septic shock (aOR=1.72, 95%CI: 1.02 to 2.91), McCabe score (life expectancy 1–5 years *vs.* >5 years: aOR=1.53, 95%CI: 0.81 to 2.54; <1 year *vs.* >5 years: aOR=7.67, 95%CI: 2.41 to 24.39; *P*=0.002), and infection with high risk bacteria for multidrug resistance (aOR=1.86, 95%CI: 1.12 to 3.09; *P*=0.016) were independently associated with mortality, but not sex (aOR for males *vs.* females=0.92, 95%CI 0.56 to 1.49; *P*=0.722). The relation between sex and mortality was not affected by age <50 years.

**Conclusions:**

The risk of mortality did not differ between sexes in patients affected by VAP. Affected males had more comorbidities, heavy alcohol use, and underwent bronchoscopy and triple antibiotic therapy more frequently.

## Introduction

Ventilator-associated pneumonia (VAP), a lung infection acquired under invasive mechanical ventilation, affects a significant proportion of intensive care unit (ICU) patients.^[^^1^^,^^2^^]^ Incidence density is 10/1000 ventilation-days to 20/1000 ventilation-days in Europe.^[^[3], [4], [5]^]^ VAP is associated with adverse outcomes, mainly prolonged mechanical ventilation and ICU stay. Attributable mortality has been estimated as around 10%, though it may widely differ according to the cause of admission to the ICU and the severity of underlying conditions.^[^^6^^]^ Sex differences were observed in VAP incidence, with males accounting for approximately two-thirds of affected patients.^[^^4^^,^^7^^,^^8^^]^ Male sex, smoking, co-morbidities (e.g., chronic lung diseases, cardiovascular diseases), severity of illness, presence of acute respiratory distress syndrome, and trauma are risk factors for VAP.^[^[8], [9], [10]^,^^11^^]^ More recently, lung dysbiosis (a disbalance in airways and lung microbiome) has been advocated as a further risk factor for VAP.^[^^12^^]^

Sex and gender are largely recognized factors of heterogeneity in the epidemiology, incidence, management, response to treatment, and prognosis of diseases.^[^^13^^,^^14^^]^ Sex (biological differences stemming from the different genetic and epigenetic background between males and females) may affect health and response to disease by various means, including differences between immune systems, hormonal milieu, cardiovascular health, susceptibility to cancer, drug pharmacokinetics, etc.^[^^13^^,^^15^^]^ Gender (a social construct referring to the attitudes, singularities, and behaviors attributed to each sex) is associated with differences in exposure, substance misuse (e.g., alcohol or tobacco), healthcare seeking behavior, and delivery of care.^[^^13^^]^ Both sex and gender can drive differences in microbiome between males and females, another potential cause of divergent health outcomes.^[^^16^^]^

We compared baseline variables, interventions, and outcomes among males and females who underwent intubation and developed VAP in the EUVAP/CAP study.^[^^17^^]^ Our objectives were to assess differences between sexes in VAP presentation, management, and prognosis and to explore if risk factors for ICU death differ between males and females.

## Methods

### Population

We performed a post hoc, not prespecified, analysis of the EUVAP/CAP cohort, a prospective, multinational, observational study designed to examine the clinical diagnosis of all pneumonia requiring mechanical ventilation and conducted in 27 ICUs from nine European countries (Belgium, France, Germany, Greece, Italy, Ireland, Portugal, Spain, and Turkey). Briefly, 2436 consecutive adults admitted to the participating ICUs during a period of 6–12 months in 2007 and 2008 were included (up to 100 per site) if they had a diagnosis of pneumonia and received invasive mechanical ventilation for at least 48 h. More details on the cohort are available elsewhere.^[^^17^^]^ The list of participating investigators and sites is reported in the Supplementary file.

The primary outcome was ICU mortality. All enrolled patients were followed until discharge from the ICU or death, whichever came first. Patients admitted for severe community-acquired pneumonia were excluded, and differences in prognosis according to biological sex have been reported elsewhere.^[^^18^^]^ For the present analysis, patients admitted to the ICU after a trauma or hospital-acquired pneumonia not fulfilling the criteria for VAP were also excluded. Strengthening the Reporting of Observational Studies in Epidemiology (STROBE) flowchart detailing distribution of eligible and excluded patients is reported as Figure 1.^[^^19^^]^

**Figure 1:**
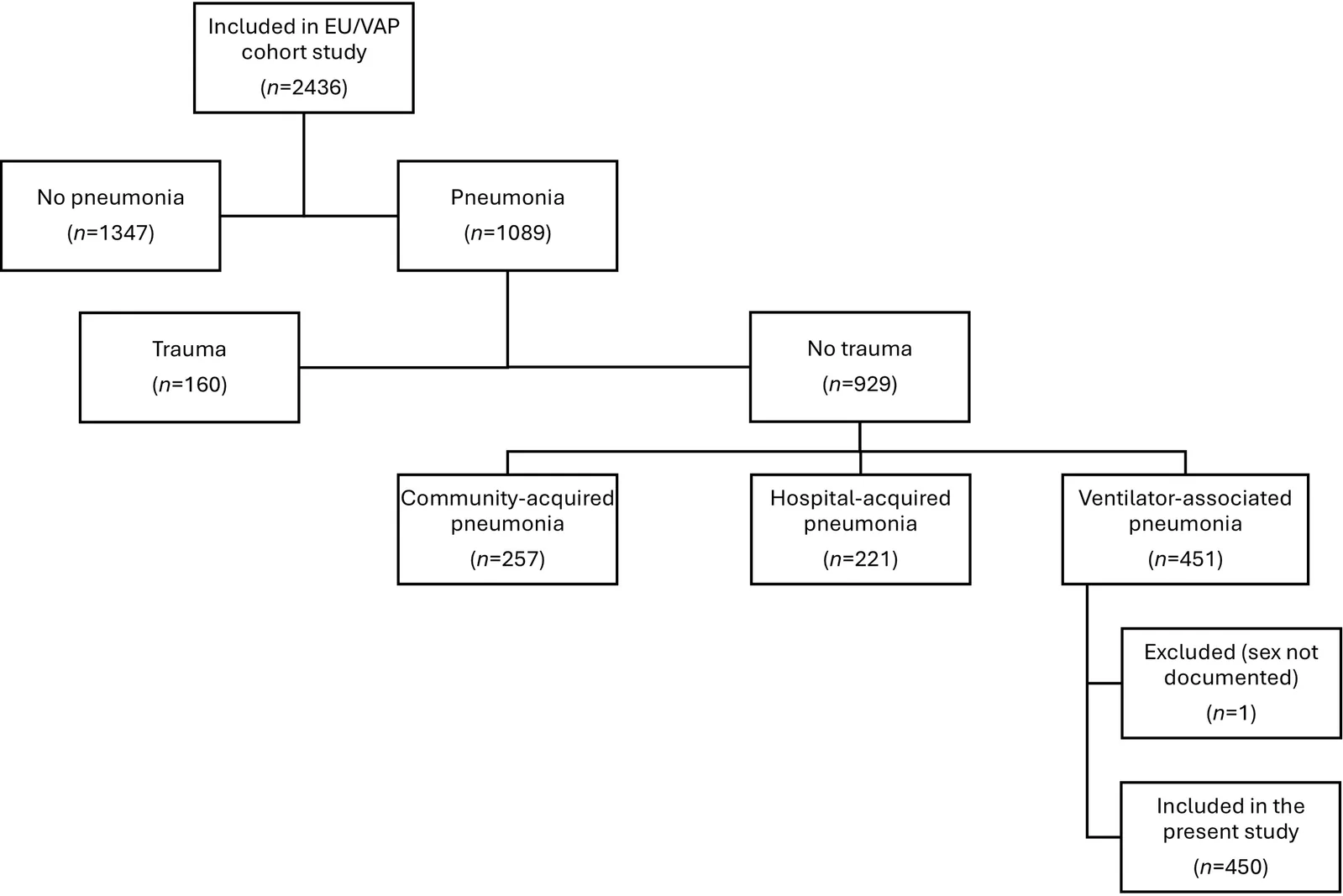
STROBE flowchart of the study.

### Definitions

Details on definitions are available elsewhere.^[^^17^^]^ VAP was defined as a suspected lung infection based on clinical and laboratory findings in a patient undergoing invasive mechanical ventilation and with compatible findings on chest X-rays. VAP was stratified by timing of occurrence. Late-onset VAP was defined as occurring 5 or more days after intubation. Patients who were intubated for community-acquired or hospital-acquired pneumonia could subsequently develop VAP, but the episode leading to intubation did not qualify for VAP.

Definite etiology was defined as a microorganism isolated from a blood or respiratory tract sample (tracheal aspirate, bronchoalveolar lavage, or protected specimen brush) and considered as definite by the physician in charge of the patient. Cultures with normal flora, *Staphylococcus epidermidis* or coagulase-negative staphylococci, and *Candida* species were considered nonpathogenic.

Sex was obtained from the medical records. No data on gender were available. Each participating center obtained ethical approval from its institution at the time of the study. Informed consent was waived because of the strictly observational nature of the study. The study has therefore been performed in accordance with the ethical standards laid down in the 1964 *Declaration of Helsinki* and its later amendments.

### Statistical analysis

We used frequencies with proportions and mean with standard deviation (SD) (normally distributed data) or median with interquartile range (IQR) (non-normally distributed data) for descriptive statistics.

Males and females were compared in univariate analysis using Fischer’s exact test, Chi-squared test, Student *t*-test (normally distributed data), or Mann–Whitney *U* test (non-normally distributed data). Survivors and nonsurvivors at ICU discharge were compared using the same tests. Variables associated with ICU death in univariate analysis with a *P* <0.05 were introduced in a multivariate logistic regression model, along with sex. Accounting for center effects was done with a fixed effect model. The analysis was then repeated separately for each sex. Heterogeneity in the relation between sex and mortality due to menopause was tested by adding an interaction term between sex and age less than 50 years in the model, the mean age at menopause being 50.5 years (95%confidence intervals [CIs]: 50.0 to 51.0) in Europe.^[^^20^^]^

A sensitivity analysis used 55 years or less as an alternative cut-off. Subgroup analyses were conducted in patients with a Simplified Acute Physiology Score (SAPS) II score of 35–58, and in patients admitted for surgery (emergent or elective), as attributable mortality may be higher in these categories of patients.^[^^6^^]^

We did not impute missing data and present complete case analyses. For descriptive purposes only, variables with more than 5% of missing data are presented but were not included in the multivariate analysis. All results are reported with 95%CI. A *P* value <0.05 was deemed significant. No adjustment was made for multiple testing. All analyses were conducted with SPSS version 25.0 (IBM Corp., Armonk, NY, USA). We adhered to Sex and Gender Equity in Research (SAGER) guidelines in the conception and reporting of this study.^[^^21^^]^

## Results

Of the 2436 patients from the initial cohort, 1089 had or developed pneumonia, among which 450 (18.5% of the initial population) fulfilled the definition of VAP and formed the population for subsequent analyses (Figure 1). Of these VAP cases, 293 (65.1%) were male, and the proportion of males included in individual hospitals ranged from 41% to 100% (Supplementary Table S1). Characteristics of the patients are displayed in Table 1. Median age was 64 years and did not differ between sexes. Septic shock was present in 32.0% of patients, and 69.8% had no fatal underlying disease (McCabe score 0). The most frequent comorbidities were chronic heart failure (28.2%) and chronic obstructive pulmonary disease (17.1%). Heavy alcohol use was significantly more frequent in males (14.3% *vs.* 6.4%, *P*=0.012). Most comorbidities were more frequent in males, though differences were not statistically significant. The category of admission (medical, scheduled, or unscheduled surgery, neurological affections) was similar. Males had been staying in the hospital for a median of 1 day before ICU admission (IQR 0–4), whereas females were predominantly admitted directly to the ICU (IQR 0–1; *P*=0.008; Table 1).

**Table 1:** Characteristics of the patients and episodes according to sex.

Characteristics	Males (*n*=293)	Females (*n*=157)	*P* value
Age (years)	64 (49–72)	65 (48–76)	0.591
<50 years	76 (25.9)	42 (26.8)	0.852
SAPS Ⅱ score	45 ± 16	46 ± 17	0.484
Patients with a SAPS Ⅱ score of 35–58	161 (55.3)	81 (52.3)	0.536
Septic shock*	89 (32.2)	45 (31.5)	0.872
McCabe score†			0.878
>5 years	199 (69.8)	104 (69.8)	
1–5 years	68 (23.9)	37 (24.8)	
<1 year	18 (6.3)	8 (5.4)	
Comorbidities			
Immunosuppression	1 (0.3)	2 (1.3)	0.247
Chronic obstructive pulmonary disease	56 (19.1)	21 (13.4)	0.124
Diabetes	47 (16.0)	27 (17.2)	0.752
Chronic heart failure	90 (30.7)	37 (23.6)	0.108
Chronic renal failure	30 (10.2)	13 (8.3)	0.501
Chronic respiratory failure	35 (11.9)	10 (6.4)	0.060
Cirrhosis	16 (5.5)	9 (5.8)	0.892
Heavy alcohol use	42 (14.3)	10 (6.4)	0.012
Non-metastatic cancer	28 (9.6)	10 (6.4)	0.247
Metastatic cancer	15 (5.1)	2 (1.3)	0.041
Hematologic cancer	1 (0.3)	2 (1.3)	0.247
Days before ICU admission‡	1 (0–4)	0 (0–1)	0.008
Timing of occurrence			0.142
Early VAP (<5 days)	83 (28.3)	55 (35.0)	
Late VAP (≥5 days)	210 (71.7)	102 (65.0)	
Diagnostic category§			0.571
Medical	170 (59.9)	93 (62.0)	
Scheduled surgery	38 (13.4)	14 (9.3)	
Unscheduled surgery	22 (7.7)	15 (10.0)	
Neurology	54 (19.0)	28 (18.7)	

Data are presented as *n* (%), mean ± standard deviation or median (interquartile range).

Missing data were due to a lack of documentation in the database. McCabe score definitions: 0, no fatal underlying disease (vital prognosis >5 years); 1, ultimately fatal (vital prognosis 1–5 years); 2, rapidly fatal (vital prognosis <1 year).

ICU, Intensive care unit; SAPS, Simplified Acute Physiology Score; VAP, Ventilator-associated pneumonia.

⁎ Data missing in 31 patients;

† Data missing in 16 patients;

‡ Data missing in 147 patients;

§ Data missing in 16 patients.

There were slight management differences between sexes: males underwent bronchoscopy more frequently (20.5% *vs.* 11.5%, *P*=0.016) and received a triple combination antibiotic therapy more frequently (33.1% *vs.* 22.3%, *P*=0.016). A definite pathogen was identified in 70.2% of patients, and 35.8% were infected by high-risk bacteria for multidrug resistance (*Pseudomonas aeruginosa*, methicillin-resistant *Staphylococcus aureus, Acinetobacter baumannii, or Stenotrophomonas* spp.; Table 2).

**Table 2:** Sex‑specific differences in pathogens, investigations, treatment, and outcomes.

Variables	Males (*n*=293)	Females (*n*=157)	*P* value
Positive blood cultures	26 (8.9)	14 (8.9)	0.849
Positive tracheal aspirate*	168 (82.8)	86 (76.8)	0.199
Positive bronchoalveolar lavage†	29 (14.2)	9 (8.0)	0.106
Positive protected specimen brush‡	12 (5.9)	3 (2.7)	0.200
Bronchoscopy	60 (20.5)	18 (11.5)	0.016
Antibiotics before ICU admission	35 (12.2)	25 (16.3)	0.222
Antibiotics in the ICU			
Monotherapy	108 (36.9)	61 (38.9)	0.677
Two initial antibiotics	185 (63.1)	96 (61.1)	0.684
Three initial antibiotics	97 (33.1)	35 (22.3)	0.016
Carbapenem use in the initial treatment	44 (15.0)	30 (19.1)	0.264
Anti-MRSA drug use in the initial treatment	25 (8.5)	10 (6.4)	0.414
Definite etiology of pneumonia	204 (69.6)	112 (71.3)	0.705
High-risk bacteria for multidrug resistance§	103 (35.2)	58 (36.9)	0.706
Polymicrobial infection	66 (22.5)	31 (19.7)	0.494
Days of mechanical ventilation (survivors)	14 (7–25)	13 (7–27)	0.695
Days in the ICU (survivors)	20 (11–33)	18 (12–42)	0.811
Death in the ICU	121 (41.3)	71 (45.2)	0.422
Death in the ICU (<50 years)	25 (32.9)	18 (42.9)	0.282

Data are presented as *n* (%), mean ± standard deviation, or median (interquartile range).

ICU, Intensive care unit; MRSA, Methicillin-resistant *Staphylococcus aureus.*

⁎ Data missing in 135 patients;

† Data missing in 133 patients, missing data were due to lack of realization of the procedure in some patients;

‡ Data missing in 136 patients;

§ *Pseudomonas aeruginosa*, MRSA, *Acinetobacter baumannii*, and *Stenotrophomonas maltophila*.

ICU mortality did not differ between males and females (41.3% *vs.* 45.2%, *P*=0.422). In patients younger than 50 years, mortality was numerically lower in males (32.9% *vs.* 42.9%), but the difference was not significant (*P*=0.282; Table 2 and Supplementary Table 2).

In survivors, days on mechanical ventilation and days in the ICU did not differ between sexes (Table 2). Older age, higher McCabe score, higher SAPS II score, presence of septic shock, definite etiology of pneumonia, polymicrobial infection or infection with high-risk bacteria for multidrug resistance, immunosuppression, and diabetes were associated with a higher risk of ICU death in the univariate analysis (Table 3). As we expected significant collinearity between comorbidities and McCabe score and between definite etiology of pneumonia and high-risk bacteria for multidrug resistance, only the latter were included in the multivariate model. Sex was finally added to the model.

**Table 3:** Patient and episode characteristics according to ICU mortality status.

Variables	Survivors (*n*=258)	Nonsurvivors (*n*=192)	*P* value
Age (years)	61 (46–72)	68 (51–75)	0.002
< 50 years	75 (29.0)	43 (22.4)	0.117
Sex (male)	172 (66.7)	121 (63.0)	0.422
SAPS II	44 ± 17	48 ± 16	0.005
Septic shock*	61 (25.7)	73 (39.9)	0.002
Definite etiology of pneumonia	169 (65.3)	147 (76.6)	0.009
Polymicrobial infection	47 (18.1)	50 (26.0)	0.044
High-risk bacteria for multidrug resistance†	69 (26.6)	92 (47.9)	<0.001
McCabe score‡			<0.001
>5 years	189 (76.8)	114 (60.3)	
1–5 years	52 (21.1)	54 (28.6)	
<1 year	5 (2.0)	21 (11.1)	
Comorbidities			
Immunosuppression	0	3 (1.6)	0.041
Chronic obstructive pulmonary disease	42 (16.2)	35 (18.2)	0.574
Diabetes	31 (12.0)	44 (22.9)	0.002
Chronic heart failure	66 (25.5)	62 (32.3)	0.113
Chronic renal failure	20 (7.7)	24 (12.5)	0.091
Chronic respiratory failure	21 (8.1)	24 (12.5)	0.124
Cirrhosis	14 (5.4)	11 (5.8)	0.871
Heavy alcohol use	27 (10.4)	25 (13.0)	0.393
Nonmetastatic cancer	19 (7.3)	19 (9.9)	0.333
Metastatic cancer	10 (3.9)	7 (3.6)	0.906
Hematologic cancer	2 (0.8)	1 (0.5)	0.745
Timing of occurrence			0.550
Early VAP (<5 days)	147 (57)	104 (52)	
Late VAP (≥5 days)	111 (43)	88 (46)	
Diagnostic category§			0.729
Medical	146 (59.3)	117 (61.9)	
Scheduled surgery	32 (13.0)	20 (10.6)	
Unscheduled surgery	23 (9.3)	14 (7.4)	
Neurology	45 (18.3)	38 (20.1)	

Data are presented as *n* (%), mean ± standard deviation, or median (interquartile range).

ICU, Intensive care unit; MRSA, Methicillin-resistant *Staphylococcus aureus*; SAPS, Simplified Acute Physiology Score; VAP, Ventilator-associated pneumonia.

⁎ Data were unavailable for 31 patients due to incomplete documentation in the database;

† *Pseudomonas aeruginosa*, MRSA, *Acinetobacter baumannii*, and *Stenotrophomonas maltophila;*

‡ Data were unavailable for 16 patients due to incomplete documentation in the database;

§ Data were unavailable for 16 patients due to incomplete documentation in the database.

In the multivariate analysis, sex was not associated with the risk of death (adjusted odds ratio [aOR] for males=0.92, 95%CI: 0.56 to 1.49, *P*=0.722; Table 4). Older age, higher McCabe score, presence of septic shock, and infection with high-risk bacteria for multidrug resistance were associated with a higher risk of death in the whole population. The interaction term between age (<50 years) and sex was not significant (*P*=0.327); similarly, it remained nonsignificant when age <55 years was used as the cut-off (*P*=0.314; Supplementary Table 2). In analyses stratified by sex, the only variable significantly associated with death in males was infection with high-risk bacteria for multidrug resistance (OR=2.56, 95%CI: 1.35 to 4.87, *P*=0.004). In females, no variable was significantly associated with death, but the association of McCabe score with mortality was almost significant (*P*=0.052 for trend). Point estimates for age (1.02 for both sexes) and septic shock (1.45 for males and 1.79 for females) were comparable to those obtained in the unstratified analysis; however, statistical significance was not retained in either sex, likely attributable to reduced statistical power following stratification.

**Table 4:** Multivariate analysis with hospital as a fixed effect.

Variables	Overall (*n*=399)		Males (*n*=266)		Females (*n*=133)	
	aOR (95%CI)	*P* value	aOR (95%CI)	*P* value	aOR (95%CI)	*P* value
Sex (ref: female)						
Male	0.92 (0.56–1.49)	0.722	-	-	-	-
Age (per 1 year)	1.02 (1.00–1.04)	0.012	1.02 (1.00–1.04)	0.097	1.02 (1.00–1.05)	0.102
SAPS II (per 1 point)	1.01 (0.99–1.02)	0.439	1.01 (0.99–1.03)	0.427	1.01 (0.98–1.04)	0.509
Septic shock	1.72 (1.02–2.91)	0.042	1.45 (0.74–2.82)	0.276	1.79 (0.65–4.92)	0.258
McCabe score (ref: >5 years)		0.002		0.065		0.052
1–5 years	1.43 (0.81–2.54)	-	1.57 (0.76–3.26)	-	1.72 (0.59–5.03)	-
<1 year	7.67 (2.41–24.39)	-	4.84 (1.20–19.56)	-	19.37 (1.57–238.46)	-
High risk bacteria for multidrug resistance*	1.86 (1.12–3.09)	0.016	2.56 (1.35–4.87)	0.004	1.31 (0.49–3.52)	0.591

Included in analysis are patients with complete data for all variables in the model (overall: 399/450; male: 266/293; female: 133/157). -: Not applicable.

aOR, Adjusted odds ratio; CI, Confidence interval; MRSA, Methicillin-resistant *Staphylococcus aureus*; SAPS, Simplified Acute Physiology Score.

⁎ *Pseudomonas aeruginosa*, MRSA, *Acinetobacter baumannii*, and *Stenotrophomonas maltophila*.

Among patients admitted following emergent or scheduled surgery, the aOR for mortality associated with male sex was 0.86 (95%CI: 0.19 to 3.97; *P*=0.845). In the subgroup with a SAPS II score of 38–54, the corresponding aOR was 0.67 (95%CI: 0.30 to 1.52; *P*=0.344).

## Discussion

In this post hoc exploratory analysis of a multicentric multinational cohort of patients affected by VAP, mortality was not affected by sex in the whole cohort, and we found no hint toward a difference before or after menopause. Males and females differed significantly regarding comorbidities, timing of ICU admission, and use of bronchoscopy or triple combination antibiotic therapy.

Of these VAP cases, 293 (65.1%) were male, consistent with results reported by others.^[^[7], [8], [9]^,^^22^^]^ Females are less frequently admitted to the ICU and less likely to receive invasive mechanical ventilation.^[^^23^^]^ Hence, this higher proportion of males partly reflects the larger population at risk of VAP. However, in a recent multicenter Italian study as well as in a recent meta-analysis, male sex was associated with an increased relative risk of VAP.^[^^4^^,^^11^^]^ This could stem from a higher prevalence of comorbidities and adverse health behaviors among males, which was apparent in our cohort.^[^^7^^,^^24^^]^

ICU mortality did not differ between sexes. This contrasts with the lower adjusted risk of mortality of males mechanically ventilated for severe community-acquired pneumonia in the EUVAP/CAP cohort (aOR=0.42), and in the higher risk of mortality of females developing VAP after trauma (aOR=1.07).^[^^18^^,^^25^^]^ The aOR for death was 1.24 in females affected by VAP in a Spanish nationwide study^[^^7^^]^ and 1.50 in a cohort study of patients who developed nosocomial infection in the ICU.^[^^26^^]^ The sex difference in the risk of mortality may be modified by age, secondary to menopausal-induced hormonal changes in females. Premenopausal females may have a lower risk of death in severe infections due to the immunomodulating effect of estrogen.^[^^27^^,^^28^^]^ We searched for a differential risk of mortality between sexes before and after 50 years. As the mean age at menopause is 50.5 years (95%CI: 50.0 to 51.0) in Europe, this cut-off should include mostly females with estrogen production.^[^^20^^]^ Crude mortality was numerically higher in females less than 50 years old, a not significant difference. The interaction term was not significant in the multivariate analysis, suggesting that the relative mortality between sexes was unaffected by this cut-off. Changing the cut-off to less than 55 years led to similar results. In contrast, recent large cohort studies have found that the adjusted OR for short-term mortality was lower for critically ill females until the age of 50–55 years but higher thereafter.^[^^28^^,^^29^^]^ However, our study had limited power to assess this issue.

Septic shock was significantly associated with mortality in the whole cohort, and the aOR was similar in both sexes. Most large recent cohorts and meta-analyses have found no difference in mortality between sexes after sepsis or septic shock,^[^[30], [31], [32]^]^ although one study has found a higher risk of death in females.^[^^33^^]^ Heterogeneity between studies in the population included geographical setting, focus of infection, completeness of risk adjustment, or timing of mortality assessment is likely an explanation for these contradictory findings. The findings of the present analysis suggest that sex by itself is not a significant risk factor for mortality in VAP, but thorough adjustment for differences between sexes in the burden of comorbidities, exposures, management in the ICU, or trajectories in the hospital is warranted.

McCabe score was independently associated with a higher risk of death. The McCabe score has rarely been used to assess the prognosis of patients with VAP. Patients with a McCabe score of 2 had a higher risk of death in the PNEUMA trial, but the variable was not retained in the logistic multivariate model.^[^^26^^]^ However, multiple comorbidities are associated with an increased risk of mortality in VAP, and it is credible that the McCabe score, which is strongly and independently associated with mortality in bloodstream infections, is also a predictor of death in VAP.^[^^4^^,^^34^^]^

Infection with multidrug-resistant bacteria was strongly associated with a higher risk of death, in the whole population and in males. The association was weaker and statistically not significant in females. Multidrug-resistance bacteria are a known risk factor for poor prognosis. A higher virulence of the pathogen, inappropriate empiric antimicrobial treatment, a high prevalence of immunosuppression and chronic respiratory diseases, and association with prolonged hospitalizations are potential explanations for this association.^[^^4^^,^^35^^]^ Some of these characteristics (chronic obstructive pulmonary disease, chronic respiratory failure, metastatic cancer, and longer hospital stay before ICU admission) were present with increased frequency in males. Additionally, the immune system of males could be less effective against some infections. Both innate and adaptive immune responses differ between sexes, secondary to hormonal differences, X-linked mosaicism, or differences in the microbiome. The overall result is an inflammatory and immune response that seems stronger in females, though the evidence that it translates into a better outcome in infectious disease is uncertain.^[^^15^^]^ Lastly, the analysis stratified by sex was underpowered, resulting in large confidence intervals. The absence of impact of multidrug-resistance bacterial infection in females could be only a chance finding and should be considered with caution.

Finally, further differences between sexes warrant comment. Males with VAP underwent bronchoscopy more often, and the initial antibiotic treatment was more often with three drugs. The proportion of pathogens (overall and multidrug resistance) identified by bronchoscopy was similar between sexes (Supplementary Table 3). These markers of more aggressive management could be related to the higher prevalence of comorbidities associated with colonization by multidrug-resistance organisms (chronic obstructive pulmonary disease and chronic respiratory failure). However, differences in management policies (conscious or unconscious), or differences in advanced directives between sexes could also be at stake.^[^^36^^]^ Some other studies also point towards less aggressive life support measures in women.^[^^23^^,^^33^^]^

The strengths of this study include the prospective multinational cohort of ventilated patients representing unselected patients admitted to academic and nonacademic hospitals. Data were collected prospectively, with few missing values, and we could adjust for most confounding factors. Our study also has limitations. Due to its multicentric and observational design, our study cohort was heterogeneous with different underlying diagnoses and management protocols. VAP diagnosis was not standardized across centers and left to the discretion of local investigators, and we lacked information on management protocols, ventilatory strategies, palliative care decisions, delirium or sedation strategies, and severity-of-illness admission scores, all of which could differ between sexes and affect the outcomes. The EUVAP/CAP database has been exclusively collected in Europe, and results are not generalizable to different geographic regions. We had no data on potential differences beyond sex (e.g., gender identity, ethnicity, languages, religion, income, and healthcare systems), which may be determinants of different management decisions. EUVAP/CAP data were collected in 2007–2008, more than 10 years before the COVID-19 pandemic, and our findings may not represent contemporary ICU management. However, biological differences between sexes are unlikely to be affected by the age of the cohort. Some subgroup analyses had low power, and the absence of association may be due to type 2 error. This is particularly relevant for females who formed only one-third of the population.

As in all observational studies, residual confounding cannot be excluded. Finally, the study was planned post hoc, with the adjusted analysis being data-driven and exploratory in nature. Therefore, our findings need confirmation in a contemporary independent cohort.

## Conclusions

Crude and adjusted mortality were not associated with sex, and we found no suggestion of an effect modification due to menopause. However, we found significant differences between sexes in the characteristics of the patients affected by VAP and in the management of the disease.

## Acknowledgements

None.

## Funding

There was no specific funding for this analysis. The original study was supported in part by 10.13039/501100002809Generalitat de Catalunya grant SGR 05/920, by CIBER Enfermedades Respiratorias (CIBERES), and by 10.13039/501100004587Carlos III Health Institute grants PI05/2410 and AI/07/90031.

## Ethics Statement

Each participating center obtained ethical approval from its institution. Informed consent was waived because of the strictly observational nature of the study. The study has therefore been performed in accordance with the ethical standards laid down in the 1964 Declaration of Helsinki and its later amendments.

## Conflict of Interest

Given their role as Editorial Board Member, Despoina Koulenti and Jordi Rello had no involvement in the peer-review of this article and has no access to information regarding its peer-review. Full responsibility for the editorial process for this article was delegated to another journal editor. The authors declare that they have no known competing financial interests or personal relationships that could have appeared to influence the work reported in this paper.

## Data Availability

The datasets analyzed during the current study are available from the corresponding author on reasonable request.

## CRediT authorship contribution statement

**Nicolas Garin:** Writing – original draft, Validation, Methodology, Formal analysis. **Despoina Koulenti:** Writing – review & editing, Validation, Investigation, Data curation. **Virginie Prendki:** Writing – review & editing. **Jordi Rello:** Writing – review & editing, Supervision, Conceptualization.

## Appendix Supplementary materials

Image, application 1
