# Supplementary material for: Ventilator-associated pneumonia in males and females: A post hoc analysis of the prospective multicenter multinational EUVAP cohort
Source: J Intensive Med. 2026 Jun 16;6(5):493–500. doi: 10.1016/j.jointm.2026.04.008 (PMC13728383; doi:10.1016/j.jointm.2026.04.008)
Supplement: Supplementary file 1 [file mmc1.docx]

**Supplementary file**

**The EUVAP/CAP Study Group members were as follows (affiliations are not current but as per the time the study was conducted):**

Djilali Annane (Raymond Poincare University Hospital, Garches, France), Rosario Amaya-Villar (Virgen del Rocio University Hospital, Seville, Spain), Apostolos Armaganidis (Attikon University Hospital, Athens, Greece), Stijn Blot (Ghent University Hospital, Ghent, Belgium), Christian Brun-Buisson (Henri Mondor University Hospital, Paris, France), Antonio Carneiro (Santo Antonio Hospital, Porto, Portugal), Maria Deja (Charite University Hospital, Berlin, Ger many), Jan DeWaele (Ghent University Hospital, Ghent, Belgium), Emili Diaz (Parc Tauli Hospital, Sabadell, Spain), George Dimopoulos (Attikon University Hospital and Sotiria Hospital, Ath ens, Greece), Silvano Cardellino (Cardinal Massaia Hospital, Asti, Italy), Jose Garnacho-Montero (Virgen del Rocio University Hospital, Seville, Spain), Muhammet Guven (Erciyes University Hos pital, Kayseri, Turkey), Apostolos Kom nos (Larisa General Hospital, Larisa, Greece), Despoina Koulenti (Attikon University Hospital, Athens, Greece and Rovira i Virgili University, Tarragona, Spain), Wolfgang Krueger (Tuebingen University Hospital, Tuebingen, Germany and Constance Hospital, Constance, Ger many), Thiago Lisboa (Joan XIII Univer sity Hospital, Tarragona, Spain and CIBER Enfermedades Respiratorias), An tonio Macor (Amedeo di Savoia Hospital, Torino, Italy), Emilpaolo Manno (Maria Vittoria Hospital, Torino, Italy), Rafael Manez (Bellvitge University Hospital, Barcelona, Spain), Brian Marsh (Mater Misericordiae University Hospital, Dub lin, Ireland), Claude Martin (Nord University Hospital, Marseille, France), Ignacio Martin-Loeches (Mater Misericordiae University Hospital, Dublin, Ireland), Pavlos Myrianthefs (KAT Hospital, Ath ens, Greece), Marc Nawynck (St Jan Hospital, Brugges, Belgium), Laurent Papa zian (Sainte Marguerite University Hospital, Marseille, France), Christian Putensen (Bonn University Hospital, Bonn, Germany), Bernard Regnier (Bichat-Claude-Bernard University Hospital, Paris, France), Jordi Rello (Joan XIII University Hospital, Tarragona, Spain), Jordi Sole-Violan (Dr Negrin Uni versity Hospital, Gran Canaria, Spain), Giuseppe Spina (Mauriziano Umberto I Hospital, Torino, Italy), Arzu Topeli (Hacettepe University Hospital, Ankara, Turkey), Hermann Wrigge (Bonn University Hospital, Bonn, Germany)

**Supplementary Table S1: Number of patients included and proportion of female, by hospital.**

| Hospital | Included patients (*n*) | Males | Females |
| --- | --- | --- | --- |
| Joan XXIII | 27 | 18 (67) | 9 (33) |
| Virgen del Rocio | 35 | 21 (60) | 14 (40) |
| Negrin Canarias | 11 | 11 (100) | 0 |
| Carneira | 12 | 9 (75) | 3 (25) |
| Kat | 15 | 7 (47) | 8 (53) |
| Komnos | 20 | 14 (70) | 6 (30) |
| Dimopoulos-Armaganidis | 18 | 14 (78) | 4 (22) |
| Topeli | 27 | 11 (41) | 16 (59) |
| Guven | 25 | 16 (64) | 9 (36) |
| Mauriziano | 13 | 9 (69) | 4 (31) |
| Maria Vittoria | 10 | 6 (60) | 4 (40) |
| Krueger | 12 | 6 (50) | 6 (50) |
| Brugges-Nawynck | 23 | 13 (57) | 10 (43) |
| Ghent-De Waale | 29 | 19 (66) | 10 (34) |
| Raymond Poincaré | 13 | 8 (62) | 5 (38) |
| Henri Mondor | 22 | 13 (59) | 9 (41) |
| Marseille Nord | 33 | 20 (61) | 13 (39) |
| Sainte Marguerite, Marseille | 4 | 3 (75) | 1 (25) |
| Claude Bernard | 2 | 2 (100) | 0 |
| Cardinal Massaja Asti | 16 | 11 (69) | 5 (31) |
| Mater Misericordiae | 9 | 8 (89) | 1 (11) |
| Bellvitge Barcelona | 16 | 12 (75) | 4 (25) |
| University Hospital Bonn | 44 | 31 (71) | 13 (29) |
| Berlin | 14 | 11 (79) | 3 (21) |

Data are presented as *n* (%).

**Supplementary Table S2: Mortality by sex in patients <50 years and <55 years.**

| Population | Mortality (male) | Mortality (female) | aOR (95%CI) |
| --- | --- | --- | --- |
| Overall | 121/293 (41.3) | 71/157 (45.2) | 0.92 (0.56–1.49) |
| <50 years | 25/76 (32.9) | 18/42 (42.9) | 0.68 (0.27–1.67) |
| <55 years | 33/94 (35.1) | 22/53 (41.5) | 0.83 (0.37–1.85) |

Data are presented as *n/N* (%). Adjusted for age, SAPS II, septic shock, McCabe score, and high-risk bacteria for multidrug resistance. aOR: Adjusted odds ratio; CI: Confidence interval.

Interaction <50 years and sex: *P*=0.327.

Interaction <55 years and sex: *P*=0.314.

**Supplementary Table S3: Pathogens identified by bronchoscopy (BAL or PSB) in males and females.**

|  | Males (60) | Females (18) | *P*-value |
| --- | --- | --- | --- |
| Definite etiology | 38 (63.3) | 11 (61.1) | 0.861 |
| High-risk bacteria for multidrug resistance | 18 (30.0) | 5 (27.8) | 0.848 |

Data are presented as *n* (%). BAL: Bronchoalveolar lavage; PSB: Protected specimen brush.
